# Piezoelectric Properties of As-Spun Poly(vinylidene Fluoride)/Multi-Walled Carbon Nanotube/Zinc Oxide Nanoparticle (PVDF/MWCNT/ZnO) Nanofibrous Films

**DOI:** 10.3390/polym16172483

**Published:** 2024-08-30

**Authors:** Lei Xu, Jiao Lv, Shengrui Yu

**Affiliations:** School of Mechanical and Electric Engineering, Jingdezhen Ceramic University, Jingdezhen 333403, China; lvj5678@163.com

**Keywords:** electrospinning, crystallinity, piezoelectricity

## Abstract

Conductive multi-walled carbon nanotubes (MWCNTs) as well as piezoelectric zinc oxide (ZnO) nanoparticles are frequently used as a single additive and dispersed in polyvinylidene fluoride (PVDF) solutions for the fabrication of piezoelectric composite films. In this study, MWCNT/ZnO binary dispersions are used as spinning liquids to fabricate composite nanofibrous films by electrospinning. Binary additives are conducive to increasing the crystallinity, piezoelectric voltage coefficient, and consequent piezoelectricity of as-spun films owing to the stretch-enhanced polarization of the electrospinning process under an applied electric field. PCZ–1.5 film (10 wt. % PVDF/0.1 wt. % MWCNTs/1.5 wt. % ZnO nanoparticles) contains the maximum β-phase content of 79.0% and the highest crystallinity of 87.9% in nanofibers. A sensor using a PCZ–1.5 film as a functional layer generates an open-circuit voltage of 10 V as it is subjected to impact loads with an amplitude of 6 mm at 10 Hz. The piezoelectric sensor reaches a power density of 0.33 μW/cm^2^ and a force sensitivity of 582 mV/N. In addition, the sensor is successfully applied to test irregular motions of a bending finger and stepping foot. The result indicates that electrospun PVDF/MWCNT/ZnO nanofibrous films are suitable for wearable devices.

## 1. Introduction

The global energy crisis, as well as environmental pollution, has been a motive to use alternative clean energy sources [1], such as solar energy [2], mechanical energy [3], and wind energy [4]. A sustainable and self-sufficient power source becomes an ideal component of intelligent electronic products and wearable devices. Growing attention is being paid to low-power and self-powered systems [5,6]. Passive piezoelectric generators deriving energy from random vibrations alleviate the current energy-shortage problem [7]. Flexible piezoelectric generators may be used in portable and wearable microdevices, such as power supplies [8], pressure sensors [9], and implantable medical devices [10].

Piezoelectric materials are essential for energy collection and conversion in piezoelectric generators. High-frequency transducers make use of piezoelectric single-crystal materials, such as quartz, without a center of symmetry because of their high stability and high-mechanical-quality factor [11]. High-power transducers utilize piezoelectric ceramics owing to their excellent piezoelectricity and high permittivity, though piezoelectric ceramics have low stability and high electrical losses. In comparison with single-crystal materials and ceramics, piezoelectric polymers have lower piezoelectric coefficients, such that they are seldom used in active transmitting transducers. In spite of this, piezoelectric polymers are often used in wearable devices as they have good flexibility and biocompatibility.

Piezoelectric polymers produce a piezoelectric effect by arranging irregular dipoles in the material under applied mechanical stretching as well as direct current polarization conditions [12,13]. Typical piezoelectric polymers include polyvinyl chloride [14], polypropylene [15], and polyvinylidene fluoride (PVDF) [16] as well as its copolymers, such as poly(vinylidene fluoride-hexafluoropropylene) (PVDF-HFP) and polyvinylidene fluoride-trifluoroethylene (PVDF-TrFE). Among them, PVDF and its copolymers are excellent candidate materials for piezoelectric generators due to their high chemical resistance [17].

PVDF contains five kinds of crystalline phases (α, β, γ, δ, and ε) [18]. Β-phase crystals have a strong, spontaneous dipole and play a dominant role in the piezoelectric, pyroelectric, and ferroelectric effects of PVDF materials. Lin et al. [19] prepared PVDF fibrous films by electrospinning, and then subjected them to hot pressing at 120 °C and 20 MPa to obtain a β-phase content as high as 89.77%. Singh et al. [20] synthesized composite films by doping transitional metal disulfide into PVDF via the hydrothermal method, in which the β-phase content increased from 54% to 80%.

Most attention is paid to composite materials by incorporating additives into polymers. Parangusan et al. [21] used the electrospinning process to fabricate PVDF-HFP/PANI-ZnS nanofibrous films with core–shell structures. The peak voltage and current reached 3 V and 1.25 μA, respectively, at 45 Hz under an applied external force of 2.5 N. Liu et al. [22] electrospun BZT-BCT/PVDF-TrFE lead-free composite nanofilms with a power density of 14.6 W/m^2^ and a force sensitivity of up to 1.5 V/N under low-pressure conditions. Li et al. [23] prepared PVDF-TrFE/ZnO composite films using the spin coating method, such that the peak voltage rose by 24.4% to 2.65 V in comparison to PVDF-TrFE films. Negar et al. [24] fabricated as-spun PVDF and lead zirconate zitanate composite films assembled into a piezoelectric sensor. The β-phase content was 70%.

Among single additives, zinc oxide (ZnO) is one of the frequently used inorganic materials as it has a high piezoelectric charge coefficient (~12.3 pC/V) [25]. The addition of ZnO to PVDF may improve the piezoelectric output. Li et al. [26] electrospun PVDF/ZnO nanorod composite membranes. From membrane-based nanogenerators, they derived an electrical throughput with an open-circuit voltage of 85 V and a short-circuit current of 2.2 μA. On the other hand, some carbon materials are often used as conductive additives. Typically, the addition of carbon nanotubes (CNTs) to PVDF may increase the charge transfer capability and β-phase PVDF content of nanofiber films. Wang et al. [27] electrospun nanofibrous films using a PVDF solution doped with multi-walled CNTs (MWCNTs). The force sensitivity reached 540 mV/N under operating conditions in the range of 0.5–5 N.

Furthermore, binary additives of piezoelectric ZnO nanoparticles and conductive CNTs are also applied to PVDFs. Many researchers prefer a casting technique for the fabrication of PVDF/ZnO/CNT composite membranes in order to obtain homogeneous membranes. Pratihar et al. [28] fabricated 15 wt. % ZnO and 0.1 wt. % MWCNT-loaded PVDF-based composite films by using a drop casting process. The film generated an a.c. output voltage of ~22 V through the application of human finger tapping, and reached a maximum power density of 21.41 μW/cm^2^ at a 4 MΩ load resistance. Kumar et al. [29] fabricated PVDF/ZnO/CNT nano-reinforced composites using the solution casting method. The composites generated a voltage peak of 1.32 V, a maximum current of 0.61 μA, and a maximum power of 0.66 μW. Khalifa et al. [30] electrospun PVDF/ZnO-decorated single-walled CNT (SWCNT) composites. The composites generated the highest voltage of 15.5 V and a power density of 8.1 μW/cm^2^ as 0.75 wt. % ZnO-decorated SWCNT/PVDF was applied to the generator.

In this work, an attempt is made to spin nanofibrous films from MWCNT/ZnO dispersions in a PVDF solution by using the electrospinning process. The dispersions are directly used as the spinning liquid. Theoretically, the binary additives may increase the crystallinity of as-spun nanofibers, owing to the stretch-enhanced polarization under the applied electric field, and consequently increase the piezoelectric effect of the as-spun nanofibrous films. On this basis, a flexible piezoelectric sensor with a PVDF/MWCNT/ZnO composite nanofibrous film as a functional layer is prepared and applied in practice.

## 2. Experiments

### 2.1. Electrospinning

**Figure 1:**
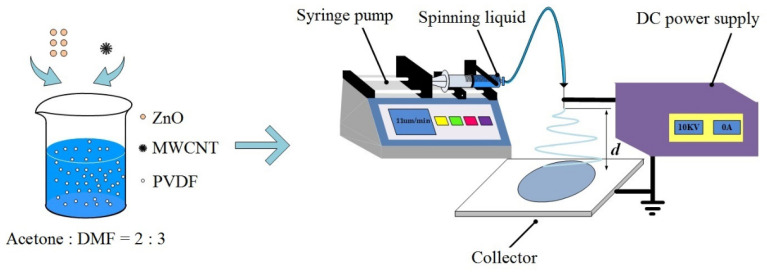
Schematic illustration of the electrospinning process.

Figure 1 shows a schematic illustration of the electrospinning process. A PVDF solution, MWCNT dispersions, and MWCNT/ZnO dispersions were prepared as the spinning liquids in advance. The electrospinning process was carried out at a dc voltage of 10 kV from a power supply (DW-P503-1ACDF0, Dongwen High-Voltage Power Supply, Tianjin, China). A metal needle with an inside diameter of 0.33 mm was connected to the positive electrode, and a plate collector was used as the ground. The needle-to-plate distance was 12 cm. A syringe full of the spinning liquid was controlled at a flow rate of 11 μL/min by a microinjection pump (LSP01-1A, Dichuang Electronic Technology, Baoding, China). Jets were whipped in space, deposited on a collector, and then became nanofibers after solvent evaporation. Every electrospinning process took 7 h to fabricate nanofibrous films. The average height of the as-spun films was about 40 μm. The as-spun films dried at 60 °C for 12 h in a drying oven (DHG-9100A, Sanfa Scientific Instrument, Shanghai, China).

### 2.2. Spinning Liquids

The applied spinning liquids included PVDF (MW = 1,000,000 g/mol, Shunjie Plastics, Shantou, China) solution, MWCNT dispersions in PVDF solution, and MWCNT/ZnO dispersions in PVDF solution. PVDF solution used a mixed solvent of acetone and N-N dimethylformamide (DMF) with a ratio of 2:3 *v*/*v.* MWCNTs (Power McLean, Shanghai, China) had an outside diameter of 40 nm, an inside diameter of 7 nm, and an average length of 15 nm. ZnO particles (Aladdin Reagent, Shanghai, China) had a diameter of 30 nm.

In order to prepare the dispersions, solid additives, i.e., MWCNTs and/or ZnO particles, were first dispersed into a mixed solvent of PVDF solution. The blend of additives and the mixed solvent was agitated for 30 min through an ultrasonic cleaning process for uniform dispersion. PVDF powders were then added into the dispersion to prepare the spinning liquid. The liquid was sealed and stirred continuously for 10 h at room temperature by a magnetic stirrer (HS-12, Qunan Experimental Instrument, Ningbo, China).

The concentration of PVDF in all of the spinning liquids was 10% in terms of mass in the mixed solvent. The neat PVDF solution without any solid additives was labeled PC–0. The MWCNT dispersions with 0.05%, 0.1%, and 0.2% MWCNTs in terms of mass in PVDF solution were labeled PC–0.05, PC–0.1, and PC–0.2, respectively. ZnO nanoparticles were then dispersed into a PC–0.1 dispersion for the fabrication of MWCNT/ZnO dispersions as the electrospun PC–0.1 film showed better piezoelectricity among MWCNT dispersions. The MWCNT/ZnO dispersions with 1.0%, 1.5%, and 2.0% ZnO particles as well as 0.1% MWCNTs in terms of mass in PVDF solution were labeled PCZ–1, PCZ–1.5, and PCZ–2. Table 1 lists the content of the spinning liquids in detail.

### 2.3. Sensor Assembly

**Figure 2:**
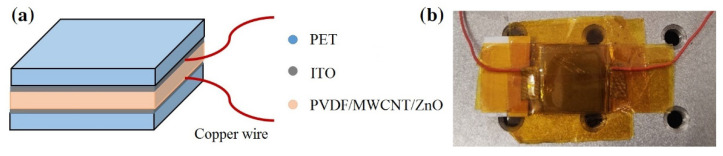
The sandwich structure of a piezoelectric sensor: (**a**) schematic diagram and (**b**) a picture of a sample on a test bench.

Figure 2 illustrates a piezoelectric sensor with a sandwich architecture. An as-spun PVDF/MWCNT/ZnO fibrous film was used as a functional layer of about 40 μm in height. Indium tin oxide (ITO) films were used as conductive layer and deposited on polyethylene terephthalate (PET) films, as shown in Figure 2a. The area of the assembled sensor was about 2 × 2 cm^2^ (Figure 2b). ITO films had a sheet resistance of 6 ohms per square, and the as-spun film was stuck to ITO films with conductive polyimide tape. Copper wires were used as lead wires for the test.

### 2.4. Characterization and Test

The morphology of as-spun nanofibrous films was observed by a scanning electron microscope (SEM). One hundred fibers were randomly selected from each film and measured by ImageJ 1.51 software. The crystalline state of PVDF material was examined by an X-ray diffractometer (XRD) and a Fourier transform infrared spectrometer (FTIR). The piezoelectricity of assembled sensors was tested under an excitation force supplied by an excitation system.

The excitation system consisted of a function generator, a power amplifier (YE5872A, Lianneng Electronic Technology, Yangzhou, China), and an electric exciter (JZK-10, Lianneng Electronic Technology, Yangzhou, China). The function generator produced wave signals, and the power amplifier enhanced the signals and drove the exciter to work. An oscilloscope (GDS-1102A-U, Guwei Electronics, Suzhou, China) was used to record the output voltage of sensors. A digital multimeter was used to measure the output current.

## 3. Results and Discussion

### 3.1. Morphological Analysis

**Figure 3:**
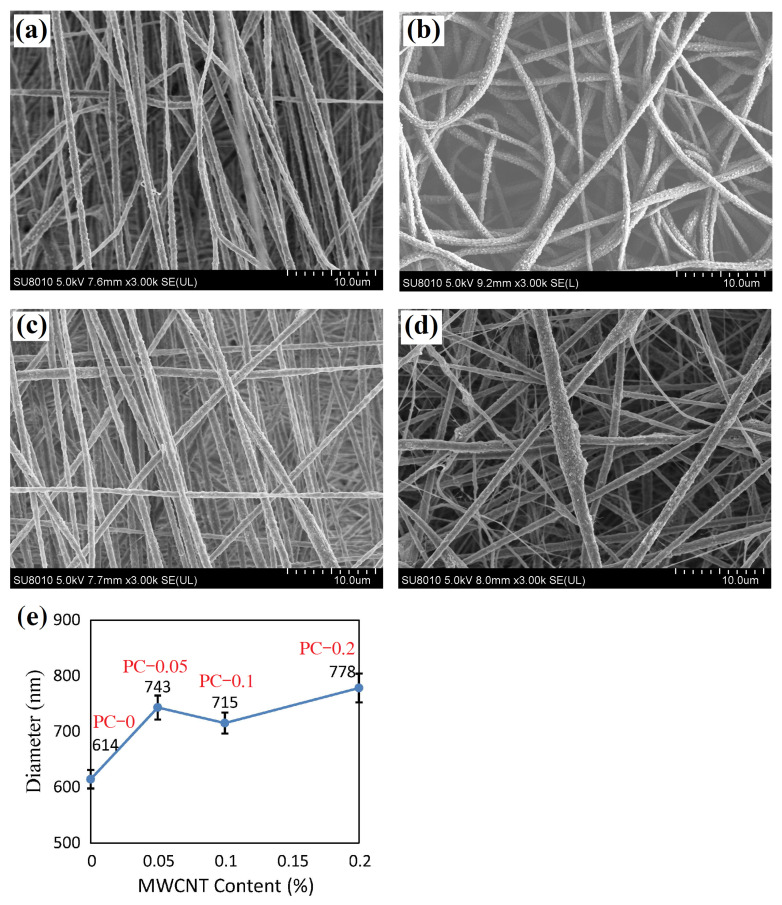
SEM images of PVDF nanofibrous film and PVDF/MWCNT composite fibrous films: (**a**) PC–0 film, (**b**) PC–0.05 film, (**c**) PC–0.1 film, (**d**) PC–0.2 film, and (**e**) average fiber diameter vs. MWCNT content.

Figure 3 shows SEM images of the neat PVDF nanofibrous film (PC–0 film) and PVDF/MWCNT composite nanofibrous films (PC–0.05, PC–0.1, and PC–0.2 films). There is no phenomenon of bead formation or adhesion into sheets [31,32]. The PC–0 film (Figure 3a) contains aligned fibers, the PC–0.05 film (Figure 3b) has uniform nanofibers, the PC–0.1 film (Figure 3c) contains mostly aligned and uniform nanofibers, and the PC–0.2 film (Figure 3d) has non-uniform nanofibers. In particular, there are nanopores on the surface of fibers in PVDF/MWCNT composite nanofibrous films. The PC–0.2 film shows lumped fibers with the most nanopores and the PC–0.1 film has the fibers with the fewest nanopores. High porosity comes from the slow volatilization rate of the solvent in PVDF solution after the deposition of nanofibers. Theoretically, high porosity may lead to the concentration of stresses and weaken the tensile strength of nanofibers.

Figure 3e characterizes the MWCNT content and average fiber diameter. The average fiber diameters are 614, 743, 715, and 778 nm, and the standard deviations are 165, 217, 189, and 257 nm for the PC–0, PC–0.05, PC–0.1, and PC–0.2 films, respectively. All of the standard errors are within 26 nm. This indicates that the addition of MWCNTs to PVDF enlarges the fibers. Among the PVDF/MWCNT nanofibrous films, the PC–0.1 film has the finest fibers with the smallest standard deviation.

**Figure 4:**
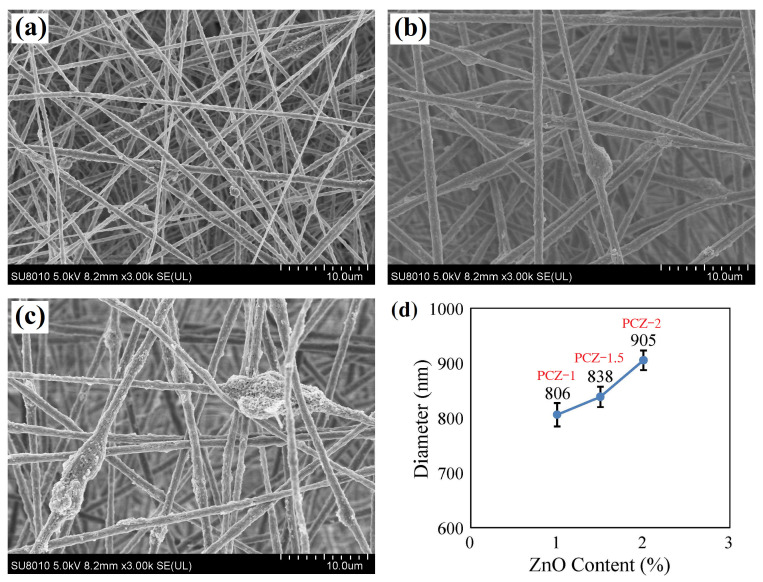
SEM images of PVDF/MWCNT/ZnO composite nanofibrous films: (**a**) PCZ–1 film, (**b**) PCZ–1.5 film, (**c**) PCZ–2 film, and (**d**) average fiber diameter vs. ZnO content.

Figure 4 demonstrates SEM images of PVDF/MWCNT/ZnO composite nanofibrous films. The PCZ–1 film contains smooth but inhomogeneous nanofibers (Figure 4a), the PCZ–1.5 film has uniform nanofibers with few lumps (Figure 4b), and the PCZ–2 film has agglomerated fibers with the most nanopores (Figure 4c). The lump and agglomeration probably result from the high surface activity of ZnO nanoparticles. Figure 4d characterizes the ZnO content and fiber diameter. The average fiber diameters are 806, 838, and 905 nm, and the standard deviations are 216, 185, and 189 nm for the PCZ–1, PCZ–1.5, and PCZ–2 films, respectively. All of the standard errors are less than 22 nm. The result represents the fact that high ZnO content results in big fibers and the agglomeration of nanoparticles under high surface activity. Although the PCZ–1 film has the finest fibers, the PCZ–1.5 film has the most homogeneous fibers among the PVDF/MWCNT/ZnO nanofibrous films.

### 3.2. Crystal Structure

**Figure 5:**
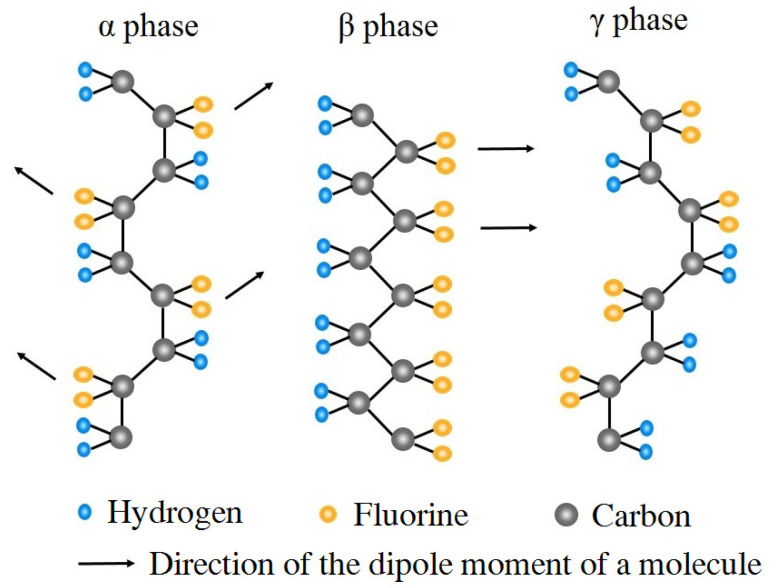
Crystalline conformations of PVDF material.

PVDF is a semi-crystalline polymer with the molecular formula –(CH_2_–CF_2_)_n_–. Among the five crystalline phases, at least four crystalline phases may transform into each other under some conditions, such as mechanical stretching and high-temperature polarization methods [33,34]. Figure 5 shows the α-, β-, and γ-phase crystalline conformations of PVDF. The α-phase crystals with the trans-gauche (TGTG) configuration have a non-polar crystal structure as molecular chains are aligned anti-parallel to each other and dipole moments counteract each other. The β-phase crystals represented by the all-trans (TTTT) configuration have strong polarity and good piezoelectricity since they have the highest dipole moment per unit cell with their dipoles pointing in the same direction. The γ-phase crystals are arranged in a polar manner by the TTTGTTTG configuration; weak polarity may be obtained by applying a high electric field.

**Figure 6:**
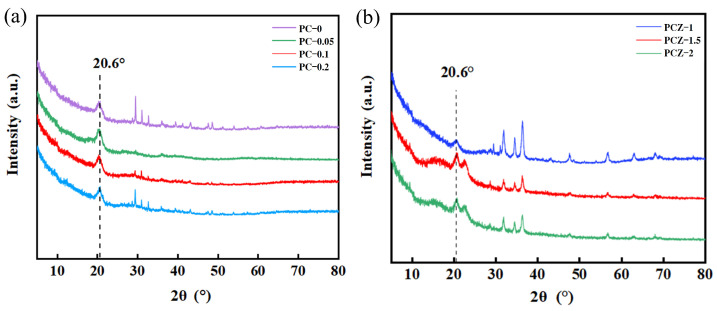
XRD patterns of various nanofibrous films: (**a**) neat PVDF film (PC–0) and PVDF/MWCNT composite films (PC–0.05, PC–0.1, and PC–0.2), and (**b**) PVDF/MWCNT/ZnO composite films (PCZ–1, PCZ–1.5, and PCZ–2).

Figure 6 shows the XRD patterns of various nanofibrous films. The neat PVDF film, PVDF/MWCNT composite films (Figure 6a), and PVDF/MWCNT/ZnO composite films (Figure 6b) exhibit a diffraction peak at 2θ = 20.6°, where the diffraction peak is the characteristic peak of the β (110) crystal plane. This indicates that all of the nanofibrous films contain β-phase PVDF crystals. This is different from PVDF powders, which mainly contain α-phase crystals with diffraction peaks at 2θ = 18.8°, 20.2°, and 26.6°, corresponding to the α (020), α (110), and α (021) crystal planes [35,36]. In addition, Figure 6b demonstrates additional characteristic peaks of ZnO at 2θ = 32.0°, 34.4°, 36.2°, 47.5°, 56.7°, 63.0°, and 68.0° for PVDF/MWCNT/ZnO nanofibrous films. These peaks are consistent with the hexagonal wurtzite structure of ZnO [37].

**Figure 7:**
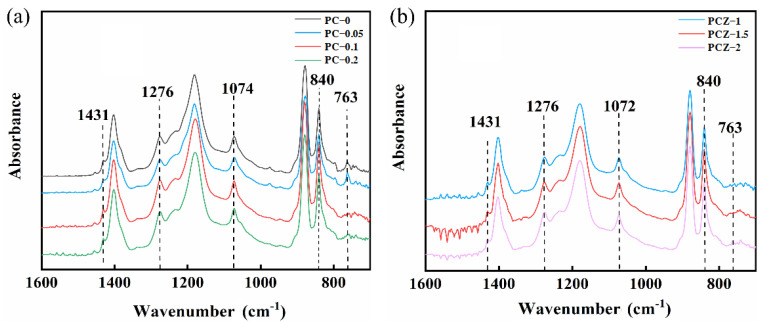
FTIR spectra of various nanofibrous films: (**a**) neat PVDF film (PC–0) and PVDF/MWCNT composite films (PC–0.05, PC–0.1, and PC–0.2), and (**b**) PVDF/MWCNT/ZnO composite films (PCZ–1, PCZ–1.5, and PCZ–2).

Furthermore, FTIR spectroscopy may also characterize molecular and crystalline structures of various as-spun films [37], as shown in Figure 7. There are peaks at 1431 cm^–1^, 1276 cm^–1^, 1074 cm^–1^, 840 cm^–1^, and 763 cm^–1^ on the FTIR spectra of the neat PVDF film and PVDF/MWCNT composite films (Figure 7a) and PVDF/MWCNT/ZnO composite films (Figure 7b). Among these peaks, the first four peaks are the peaks for β-phase PVDF crystals, while the last peak is the exclusive peak for α-phase PVDF crystals. All of the FTIR spectra show strong β-phase peaks and weak α-phase peaks. Hence, the electrospinning process stimulates a transition from α-phase to β-phase crystals, since electrospinning liquids in space experience extreme mechanical stretching and electric polarization under applied electric potential.

To characterize the fraction of piezoelectric crystalline regions, the crystallinity, *F_β_*, of β-phase PVDF crystals [38,39] is defined as follows:

(1) $$F_{\beta }=\frac{A_{\beta }}{\left(\frac{K_{\beta }}{K_{\alpha }}\right)A_{\alpha }+A_{\beta }},$$

where *K_α_* = 6.1 × 10^4^ cm^2^/mol is the absorption coefficient of α-phase PVDF crystals at the absorption peak, *K_β_* = 7.7 × 10^4^ cm^2^/mol is the absorption coefficient of β-phase PVDF crystals at the absorption peak, A_α_ is the absorption peak intensity of α-phase PVDF crystals at 763 cm^–1^, and *A_β_* is the absorption peak intensity of β-phase PVDF crystals at 840 cm^–1^. Table 2 lists the β-phase content and the crystallinity of PVDF in various nanofibrous films. By the Jade fitting, the PC–0 film contains β-phase content of 76.6% and crystallinity of 42.7%, the PC–0.1 film contains the maximum β-phase content of 77.4% and the highest crystallinity of 70.4% among PVDF/MWCNT composite films, and the PCZ–1.5 film contains the maximum β-phase content of 79.0% and the highest crystallinity of 87.9% among PVDF/MWCNT/ZnO composite films. The result indicates that the addition of MWCNTs and ZnO nanoparticles enhance the crystallinity of PVDF composite films. Table 3 lists the crystalline parameters and output power of PVDF composite films extracted from recent references. In comparison with these references, the present work shows that as-spun composite nanofibrous films have higher crystallinity.

However, it seems that the β-phase PVDF content slightly varies with the addition of MWCNTs and ZnO particles in comparison with the neat PVDF nanofibrous film. In spite of this, the transition efficiency of α→β-phase PVDF crystals benefits from appropriate MWCNT content. MWCNTs construct a conductive network in a polymer matrix. As MWCNTs disperse uniformly in the polymer matrix, polymer molecules may fully encapsulate carbon nanotubes. The interfacial interaction presents between the surface charges of MWCNTs and -CH_2_- dipoles of PVDF molecular chains [41]. MWCNTs increase interfacial interactions such that electrospun nanofibers produce a strong electrostriction effect under an applied electric field. The electrostriction effect promotes the nucleation of β-phase crystals during electrospinning. Few MWCNTs may also result in inhomogeneous MWCNT distribution in electrospun nanofibers (Figure 3b), while excessive MWCNTs cause lumps of nanofibers with many nanopores (Figure 3d). Both cases weaken the tensile strength and electrical conductivity of electrospun nanofibers.

The transition of PVDF crystals from the α phase to the β phase may then also benefit from appropriate ZnO content in nanofibrous films. This is attributed to the interaction between positive charges carried by the CH_2_ group of PVDF molecular chains and negative charges carried by ZnO nanoparticles. The CH_2_ groups in PVDF molecular chains are regularly arranged on the surface of piezoelectric ZnO particles increasing the β-phase content of PVDF [42].

### 3.3. Piezoelectricity

**Figure 8:**
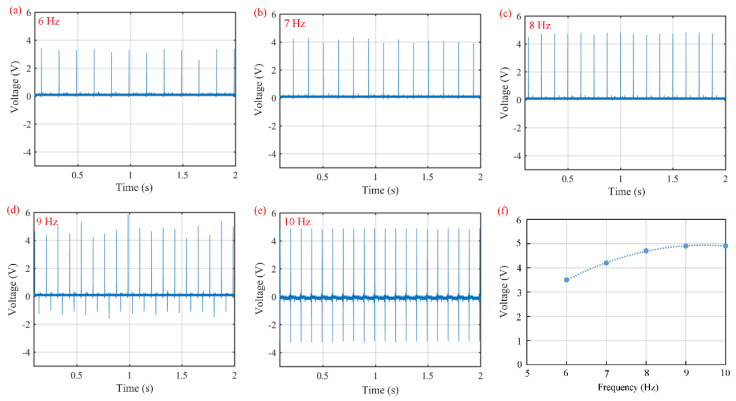
Open-circuit voltages of piezoelectric sensors with the PC–0.1 film as a functional film under applied impact loads at various excitation frequencies: (**a**) 6 Hz, (**b**) 7 Hz, (**c**) 8 Hz, (**d**) 9 Hz, (**e**) 10 Hz, and (**f**) open-circuit voltage vs. excitation frequency.

Figure 8 shows the open-circuit voltage of piezoelectric sensors using the PC–0.1 film as a functional layer under applied impact loads at various excitation frequencies. The no-load amplitude is 6 mm. Piezoelectric sensors output forward pulse signals at compressed states and negative pulse signals at released states. The average open-circuit voltage is 3.5 V for 6 Hz (Figure 8a), 4.2 V for 7 Hz (Figure 8b), 4.7 V for 8 Hz (Figure 8c), 4.9 V for 9 Hz (Figure 8d), and 4.9 V for 10 Hz (Figure 8e). Especially at 9 Hz, the open-circuit voltage is unstable and varies between 4.2 V and 5.8 V. It is believed that the piezoelectric sensor works at its mechanically natural frequency.

The relationship between open-circuit voltage and excitation frequency is illustrated in Figure 8f. Below the natural frequency, the open-circuit voltage increases with increasing excitation frequency, as higher excitation frequency leads to faster electric polarization and more polarized charges. The accumulation of polarized charges raises the open-circuit voltage. As the excitation frequency exceeds the natural frequency, the open-circuit voltage arrives at a stable value, i.e., 4.9 V.

**Figure 9:**
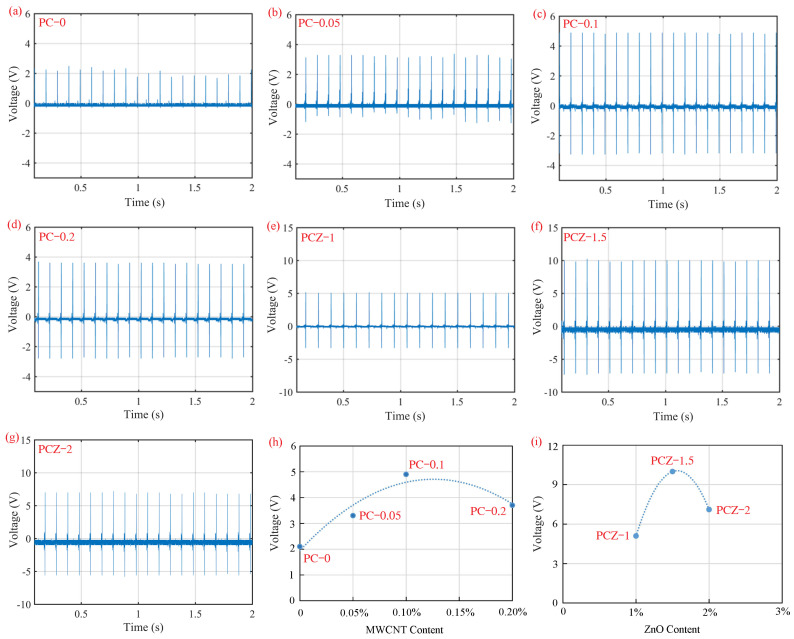
Open-circuit voltages of piezoelectric sensors with various nanofibrous films as functional layers under applied impact loads at 10 Hz: (**a**) PC–0 film, (**b**) PC–0.05 film, (**c**) PC–0.1 film, (**d**) PC–0.2 film, (**e**) PCZ–1 film, (**f**) PCZ–1.5 film, (**g**) PCZ–2 film, (**h**) open-circuit voltage vs. MWCNT content, and (**i**) open-circuit voltage vs. ZnO content.

Figure 9 demonstrates the open-circuit voltage of piezoelectric sensors using the neat PVDF nanofibrous film, PVDF/MWCNT composite nanofibrous films, and PVDF/MWCNT/ZnO composite nanofibrous films as functional layers. All of the sensors are subjected to impact loads with a no-load amplitude of 6 mm at 10 Hz, which is above the natural frequency. The open-circuit voltage is 2.1 V for the PC–0 film (Figure 9a), 3.3 V for the PC–0.05 film (Figure 9b), 4.9 V for the PC–0.1 film (Figure 9c), 3.7 V for the PC–0.2 film (Figure 9d), 5.1 V for the PCZ–1 film (Figure 9e), 10 V for the PCZ–1.5 film (Figure 9f), and 7.1 V for the PCZ–2 film (Figure 9g).

The effect of MWCNT content in PVDF/MWCNT composite nanofibrous films on open-circuit voltage is characterized by Figure 9h. All of the PVDF/MWCNT composite nanofibrous films (PC–0.05, PC–0.1, and PC–0.2 films) output higher open-circuit voltage than the neat PVDF film (PC–0 film). MWCNTs effectively compose a conductive circuit to reduce the internal resistance of sensors [43], such that the dispersion of MWCNTs promotes the piezoelectricity of PVDF nanofibrous films. Among PVDF/MWCNT composite nanofibrous films, the PC–0.1 film has the highest open-circuit voltage of 4.9 V owing to its high β-phase content and crystallinity of PVDF.

The effect of ZnO content in PVDF/MWCNT/ZnO composite nanofibrous films on open-circuit voltage is characterized by Figure 9i. In comparison with the neat PVDF film and PVDF/MWCNT films, PVDF/MWCNT/ZnO films output higher open-circuit voltage. This indicates that the addition of ZnO particles promotes the piezoelectricity of nanofibrous films, since ZnO particles themselves are a piezoelectric material. The hexagonal zinc fiber structure of ZnO is conducive to the conversion of mechanical energy into electrical energy owing to a low dielectric coefficient. Among all of the nanofibrous films, the PCZ–1.5 film shows the highest open-circuit voltage of 10 V because of its maximum β-phase content and highest crystallinity of PVDF.

On the other hand, the output voltage, *V* [16], may be characterized as follows:

(2) $$V=g_{33}\sigma h,\;$$

where *g*_33_ is the piezoelectric voltage coefficient and defined by the piezoelectric charge coefficient, *d*_33_, divided by the relative permittivity of as-spun films; *σ* is the applied stress; and *h* is the thickness of the films. In this work, the applied stress is constant as the electric exciter sets to a constant. The thickness of as-spun films is approximately 40 μm under the same electrospinning process. In this case, the output voltage represents the piezoelectric voltage coefficient, *g*_33_. Thus, the addition of MWCNTs and ZnO nanoparticles into PVDF promotes the piezoelectric voltage coefficient, *g*_33_, of PVDF nanofibrous films. The PC–0.1 film has the highest piezoelectric voltage coefficient among PVDF/MWCNT composite films, while the PCZ–1.5 film has the highest piezoelectric voltage coefficient among PVDF/MWCNT/ZnO composite films.

**Figure 10:**
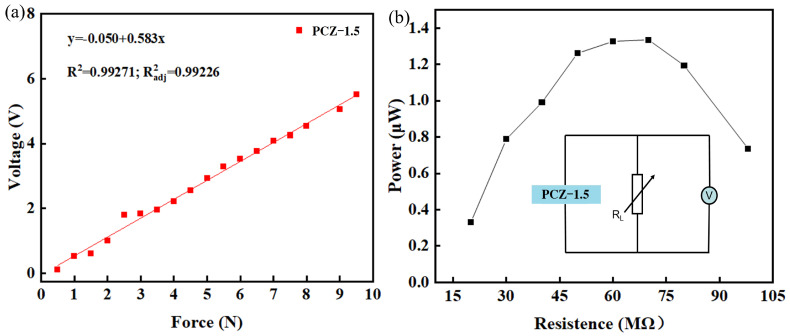
Piezoelectric performance of piezoelectric sensors with applied PCZ–1.5 film as a functional layer: (**a**) force sensitivity and (**b**) output power. The insert shows a circuit with which to measure the output power.

Furthermore, the PCZ–1.5 film is subjected to more performance tests as a consequence of its excellent piezoelectricity. Figure 10a characterizes its open-circuit voltage and external impact force. The output voltage linearly increases from 0.12 V to 2.72 V as the applied impact force increases from 0.5 N to 5 N. By the slope of the voltage–force line, the piezoelectric force sensitivity is 582 mV/N.

Figure 10b demonstrates the output power of piezoelectric sensors with applied PCZ–1.5 film as the functional layer. The insert shows a circuit with which to measure the output power via a variable resistor. The current is 0.6 μA and the voltage is 2.2 V when the resistance is set to 70 MΩ. At this point, the maximum output power is 1.32 μW. The power density, defined as the power divided by the area of the PCZ–1.5 film, is 0.33 μW/cm^2^.

**Figure 11:**
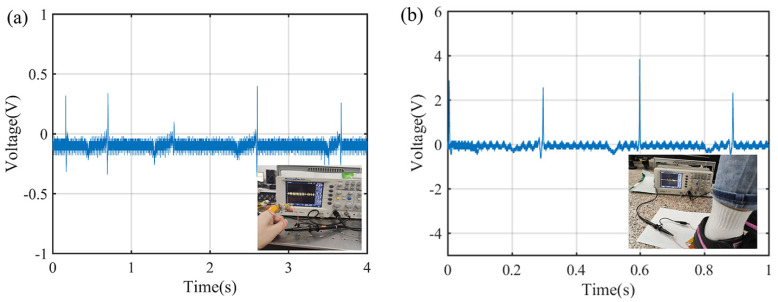
The movement detection of a human body by using the PCZ–1.5 film as a functional layer of a piezoelectric sensor: (**a**) the output voltage of a bending finger at the joint and (**b**) the output voltage of stepping a foot on the sole.

More applications of the piezoelectric sensor with the PCZ–1.5 film are made to test the irregular motion of a human body, as shown in Figure 11a. The maximum voltage is 0.03 V for bending a finger at the joint and 2 V for stepping a foot on the sole, as shown in Figure 11b. The result indicates that PVDF/MWCNT/ZnO composite nanofibrous films are suitable for wearable devices.

## 4. Conclusions

PVDF/MWCNT/ZnO nanofibrous films are directly electrospun from MWCNT/ZnO dispersion in PVDF solution. In comparison with neat PVDF nanofibrous films, the addition of MWCNTs and ZnO nanoparticles gives rise to some of the following changes in properties:

- Morphologically, binary additives make nanofibers bigger. A moderate amount of binary additives may produce aligned and homogeneous nanofibers through the electrospinning process. The MWCNT content results in many nanopores left on a nanofiber’s surface after a slow volatilization of solvent. The ZnO content leads to agglomeration owing to the high surface activity of ZnO nanoparticles.
- Crystallographically, binary additives enhance crystallinity, but they slightly change the β-phase PVDF content. Naturally, the formation of β-phase PVDF crystals is dependent on the effect of stretch-enhanced polarization from the electrospinning process. Moderate amounts of MWCNTs and ZnO nanoparticles are conducive to obtain high crystallinity and high β-phase content.
- Piezoelectrically, binary additives promote the output voltage and piezoelectric voltage coefficient, since MWCNTs may construct a conductive network in the PVDF matrix and ZnO nanoparticles themselves are a piezoelectric material.

In this work, the electrospun PCZ–1.5 film shows the highest piezoelectricity among various as-spun composite nanofibrous films. A piezoelectric sensor using the PCZ–1.5 film as the functional layer reaches a sensitivity of 582 mV/N and a power density of 0.33 μW/cm^2^. The sensor successfully tests the irregular motions of a bending finger and a stepping foot; it represents the potential applications in self-power energy systems and low-power wearable devices.

## Figures and Tables

**Table 1:** The content of various spinning liquids.

Spinning Liquid	Solvent	Solute in Solvent (by Mass)	Additives in Solvent (by Mass)
PC–0	Acetone–DMF (40:60 vol %)	10% PVDF	None
PC–0.05			0.05% MWCNTs
PC–0.1			0.1% MWCNTs
PC–0.2			0.2% MWCNTs
PCZ–1			0.1% MWCNTs and 1.0% ZnO
PCZ–1.5			0.1% MWCNTs and 1.5% ZnO
PCZ–2			0.1% MWCNTs and 2.0% ZnO

**Table 2:** The β-phase content and crystallinity of PVDF in various nanofibrous films.

Film	B-Phase Content (%)	Crystallinity (%)
PC–0	76.6	42.7
PC–0.05	67.3	55.5
PC–0.1	77.4	70.4
PC–0.2	69.7	51.4
PCZ–1	73.8	67.5
PCZ–1.5	79.0	87.9
PCZ–2	77.1	67.0

**Table 3:** The crystalline parameters and output power of PVDF composite films extracted from recent references.

Solution	Additives	Fabrication	B-Phase Content	Crystallinity	Output Power	Reference
10 wt % PVDF	5 wt % MWCNTs	Electrospinning	68.4%	38.1%	81.8 nW for charging a capacitor	[16]
22 wt % PVDF	5 wt % ZnO	Electrospinning	/	/	10 μW/cm^2^ under impact loads of 5 N at 0.4 Hz	[40]
1.35 g PVDF in 10 mL of solvent	0.75 wt % ZnO-decorated SWCNTs	Electrospinning	95%	36.1%	8.1 μW/cm^2^ at 10 MΩ load resistance	[30]
8 wt % PVDF	0.1 wt % MWCNTs, 15 wt % ZnO	Drop casting	88.9% (Electroactive phase)	32.4%	21.41 μW/cm^2^ at 4 MΩ load resistance	[28]
2 g PVDF in 10 mL of solvent	1.5 wt % CNTs, 15 wt % ZnO	Solution casting	62%	51.16%	0.66 μW for bending of legs, arms, and wrist	[29]

## Data Availability

The raw data supporting the conclusions of this article will be made available by the authors upon request.

## References

[1] Qureshy A.M.M.I., Dincer I. (2020). A new integrated renewable energy system for clean electricity and hydrogen fuel production. Int. J. Hydrog. Energy.

[2] Xu W., Huang L.B., Wong M.C., Chen L., Bai G., Hao J. (2017). Environmentally friendly hydrogel-based triboelectric nanogenerators for versatile energy harvesting and self-powered sensors. Adv. Energy Mater..

[3] Yoo H.G., Byun M., Jeong C.K., Lee K.J. (2015). Performance enhancement of electronic and energy devices via block copolymer self-assembly. Adv. Mater..

[4] Zhang K., Wang S., Yang Y. (2017). A One-structure-based piezo-tribo-pyro-photoelectric effects coupled nanogenerator for simultaneously scavenging mechanical, thermal, and solar energies. Adv. Energy Mater..

[5] Dong K., Wang Y.C., Deng J., Dai Y., Zhang S.L., Zou H., Gu B., Sun B., Wang Z.L. (2017). A highly stretchable and washable all-yarn-based self-charging knitting power textile composed of fiber triboelectric nanogenerators and supercapacitors. Acs Nano.

[6] Lai Y.C., Deng J., Zhang S.L., Niu S., Guo H., Wang Z.L. (2017). Single-thread-based wearable and highly stretchable triboelectric nanogenerators and their applications in cloth-based self-powered human-interactive and biomedical sensing. Adv. Funct. Mater..

[7] Yuan H., Lei T., Qin Y., Yang R. (2019). Flexible electronic skins based on piezoelectric nanogenerators and piezotronics. Nano Energy.

[8] He Z., Mohsenzadeh E., Zhang S., Rault F., Salaün F. (2023). Development of high-sensitive piezoelectric nanogenerators of all-organic PVDF multilayer nanofibrous membrane with innovative 3D structure via electrohydrodynamic processes. J. Polym. Res..

[9] Wang N., Dou W., Hao S., Cheng Y., Zhou D., Huang X., Jiang C., Cao X. (2019). Tactile sensor from self-chargeable piezoelectric supercapacitor. Nano Energy.

[10] Feng H., Zhao C., Tan P., Liu R., Chen X., Li Z. (2018). Nanogenerator for biomedical applications. Adv. Healthc. Mater..

[11] Chen Y., Zhang Y., Zhang L., Ding F., Schmidt O.G. (2017). Scalable single crystalline PMN-PT nanobelts sculpted from bulk for energy harvesting. Nano Energy.

[12] He Z., Rault F., Lewandowski M., Mohsenzadeh E., Salaün F. (2021). Electrospun PVDF nanofibers for piezoelectric applications: A review of the influence of electrospinning parameters on the β phase and crystallinity enhancement. Polymers.

[13] He Z., Rault F., Vishwakarma A., Mohsenzadeh E., Salaün F. (2022). High-aligned PVDF nanofibers with a high electroactive phase prepared by systematically optimizing the solution property and process parameters of electrospinning. Coatings.

[14] Zhang P., Zhang W., Zhang H. (2021). A triboelectric nanogenerator based on waste Polyvinyl chloride for Morse code generator. Sens. Actuators A Phys..

[15] Martins P., Lopes A.C., Lanceros-Mendez S. (2014). Electroactive phases of poly(vinylidene fluoride): Determination, processing and applications. Prog. Polym. Sci..

[16] Yu H., Huang T., Lu M., Mao M., Zhang Q., Wang H. (2013). Enhanced power output of an electrospun PVDF/MWCNTs-based nanogenerator by tuning its conductivity. Nanotechnology.

[17] Ruan L.X., Yao X.N., Chang Y.F., Zhou L., Qin G., Zhang X. (2018). Properties and applications of the β phase poly(vinylidene fluoride). Polymers.

[18] Cui Z., Hassankiadeh N.T., Zhuang Y., Drioli E., Lee Y.M. (2015). Crystalline polymorphism in poly(vinylidenefluoride) membranes. Prog. Polym. Sci..

[19] Lin B., Chen G.D., He F.A., Li Y., Yang Y., Shi B., Feng F.-R., Chen S.-Y., Lam K.-H. (2023). Preparation of MWCNTs/PVDF composites with high-content β form crystalline of PVDF and enhanced dielectric constant by electrospinning-hot pressing method. Diam. Relat. Mater..

[20] Singh V., Singh B. (2020). Fabrication of PVDF-transition metal dichalcogenides based flexible piezoelectric Nanogenerator for energy harvesting applications. Mater. Today Proc..

[21] Parangusan H., Bhadra J., Al-Thani N. (2021). Flexible piezoelectric nanogenerator based on [P(VDF-HFP)]/ PANI-ZnS electrospun nanofibers for electrical energy harvesting. J. Mater. Sci-Mater. El.

[22] Liu J., Yang B., Lu L., Wang X., Li X., Chen X., Liu J. (2020). Flexible and lead-free piezoelectric nanogenerator as self-powered sensor based on electrospinning BZT-BCT/P(VDF-TrFE) nanofibers. Sens. Actuat. A-Phys..

[23] Li J., Zhao C., Xia K., Liu X., Li D., Han J. (2019). Enhanced piezoelectric output of the PVDF-TrFE/ZnO flexible piezoelectric nanogenerator by surface modification. Appl. Surf. Sci..

[24] Nega C., Ramin K., Ali A.Y., Rashidi A., Golestanifard F. (2020). A flexible piezoelectric pressure sensor based on PVDF nanocomposite fibers doped with PZT particles for energy harvesting applications. Ceram. Int..

[25] Zhang M., Liu C., Li B., Shen Y., Wang H., Ji K., Mao X., Wei L., Sun R., Zhou F. (2023). Electrospun PVDF-based piezoelectric nanofibers: Mate-rials, structures, and applications. Nanoscale Adv..

[26] Li J., Chen S., Liu W., Fu R., Tu S., Zhao Y., Dong L., Yan B., Gu Y. (2019). High performance piezoelectric nanogenerators based on electrospun ZnO nanorods/poly(vinylidene fluoride) composite membranes. J. Phys. Chem. C.

[27] Wang A., Hu M., Zhou L., Qiang X. (2018). Self-Powered wearable pressure sensors with enhanced piezoelectric properties of aligned P(VDF-TrFE)/MWCNT composites for monitoring human physiological and muscle motion signs. Nanomaterials.

[28] Pratihar S., Patra A., Sasmal A., Medda S.K., Sen S. (2021). Enhanced dielectric, ferroelectric, energy storage and mechanical energy harvesting performance of ZnO–PVDF composites induced by MWCNTs as an additive third phase. Soft Matter.

[29] Kumar A., Jaiswal S., Joshi R., Yadav S., Dubey A., Sharma D., Lahiri D., Lahiri I. (2023). Energy harvesting by piezoelectric polyvinylidene fluoride/zinc oxide/carbon nanotubes composite under cyclic uniaxial tensile deformation. Polym. Compos..

[30] Khalifa M., Peravali S., Varsha S., Anandhan S. (2022). Piezoelectric energy harvesting using flexible self-poled electroactive nanofabrics based on PVDF/ZnO-decorated SWCNT nanocomposites. JOM.

[31] Reneker D.H., Yarin A.L. (2008). Electrospinning jets and polymer nanofibers. Polymer.

[32] Li D., Xia Y.N. (2004). Electrospinning of nanofibers: Reinventing the wheel?. Adv. Mater..

[33] Abolhasani M.M., Shirvanimoghaddam K., Naebe M. (2017). PVDF/graphene composite nanofibers with enhanced piezoelectric performance for development of robust nanogenerators. Compos. Sci. Technol..

[34] Gregorio R.G. (2010). Determination of the α, β, and γ crystalline phases of poly(vinylidene fluoride) films prepared at different conditions. J. Appl. Polym. Sci..

[35] Kabir E., Khatun M., Nasrin L., Raihan M.J., Rahman M. (2017). Pure β-phase formation in polyvinylidene uoride (PVDF)-carbon nanotube composites. J. Phys. D Appl. Phys..

[36] Mohamadi S., Sharifi-Sanjani N. (2016). Crystallization of PVDF in graphene-filled electrospun PVDF/PMMA nanofibers processed at three different conditions. Fiber Polym..

[37] Singh H.H., Khare N. (2018). Flexible ZnO-PVDF/PTFE based piezo-tribo hybrid nanogenerator. Nano Energy.

[38] Yousefi A.A. (2011). Influence of polymer blending on crystalline structure of polyvinylidene fluoride. Iran. Polym. J..

[39] Salimi A., Yousefi A.A. (2003). FTIR studies of beta-phase crystal formation in stretched PVDF films. Polym. Test..

[40] Li G.Y., Zhang H.D., Guo K., Ma X.S., Long Y.Z. (2020). Fabrication and piezoelectric-pyroelectric properties of electrospun PVDF/ZnO composite fibers. Mater. Res. Express.

[41] Mahanty B., Ghosh S.K., Maity K., Roy K., Sarkar S., Mandal D. (2021). All-fiber pyro- and piezo-electric nanogenerator for IoT based self-powered health-care monitoring. Mater. Adv..

[42] Thakur P., Kool A., Hoque N.A., Bagchi B., Khatun F., Biswas P., Brahma D., Roy S., Banerjee S., Das S. (2017). Superior performances of in situ synthesized ZnO/PVDF thin film based self-poled piezoelectric nanogenerator and self-charged photo-power bank with high durability. Nano Energy.

[43] Sun H., Tian H., Yang Y. (2013). A novel flexible nanogenerator made of ZnO nanoparticles and multiwall carbon nanotube. Nanoscale.

